# Thanks to Reviewers

**DOI:** 10.1177/21514593261428619

**Published:** 2026-04-17

The journal sincerely thanks the following individuals who reviewed one or more manuscripts during 2025:

Abboud, Johnny

Abdallat, Anas Al

Adler, Rachel R.

Alhossan, Abdullah M.

Antunes, Douglas Eulálio

Arcidiacono, Gaetano Paride

Aygün, Ümit

Azizan, Azliyana

Babst, Reto

Basile, Giuseppe

Beaupre, Lauren

Becoja Dpm, Besim

Belen’Kiy, Igor’ G.

Ben Bouzid, Yassine

Berkay Girgin, Ahmet

Bernardo-Filho, Mario

Bhadani, Janki Sharan

Boardman, Douglas

Borrelli Jr., Joseph

Bulut, Halil

Busel, Gennadiy

Cai, Zhen-Cun

Cedeno-Veloz, Bernardo Abel

Çevik, Hüsna Sarıca

Chabungbam, Margaret

Chan, Rachel

Charalambous, Charalambos P.

Chen, Yu-Pin

Cnossen, J.D.

Coscia, Atticus

Cotterell, Ilvy

Cudejko, Tomasz

Curiel, Ivette T

Declercq, Madeleine Grace

Demers, Alex J.

Dengler, Julius

Drinkwater, Christopher

Du, Yaoqiang

Durdu, Habibe

Eardley, Will

Elbaseet, Hesham Mohamed

Emir, Sevde Nur

Endo, Kaori

Erika De Melo Marinho, Patrícia

Essien, Ubong Ekanem

Faisal, Tanvir R.

Ferris, Helena A.

Frankenhoff, Jessica

Geurts, Jeroen

Ghazwan, Aseel

Gholipour, Morteza

Godde, K

Godoy Monzón, Daniel

Goh, En Lin

González De Villaumbrosia, Cristina

Habbous, Steven

Hagen, Jennifer

Haller, Justin M.

Han, Xiaoguang

Harris, A. H. S.

Hashimoto, Kazuhiko

He, Keqiang

Henken, Espen

Hernández-Pascual, Carlos

Hill, J Ryan

Horoz, Levent

Hou, Zhiyong

Hourlier, Hervé

Ibrahim, Mariam

Iida, Hiroki

Ishida, Shinnosuke

Jain, Kamal

Jaja, Promise Tamunoipriala

Jarusriwanna, Atthakorn

Jengojan, Suren

Jin, Wu

Jodoin, Zachary

Kalan Farma, Khadijeh

Kanakis, Ioannis

Kannan, Venkatesan Senthil

Karaduman, Zekeriya Okan

Kaya, Salih

Kazez, Muhammed

Kjærvik, Cato

Koeppe, Jeanette

Kon, Kam King Charles

Kuang, Lei

Kumar, Ranjith

Laletin, Vladimir

Lamberti, Edgar Manuel Bodu

Larsen, Michael Houlind

Layon, Daniel

Lazar Mioc, Mihail

Lee, Tae Sung

Lee, Glenn

Leicht, Hanna

Levine, Arielle Richey

Li, Lian-Hua

Lim, Jae-Young

Liu, Bingli

Liu, Huanqiu

Liu, Jiayong

Loh, Bryan

Lu, Shibao

Magaziner, Jay

Martadiani, Elysanti

Mcnicoll, Lynn

Mears, Dana

Mekhael, Elio

Mendelson, Daniel

Mendelson, Daniel Ari

Merriman, Niamh A.

Misca, Liviu-Coriolan

Mizoguchi, Yasuaki

Mohd Nawi, Siti Nurbaya

Molina-Olivella, Guillem

Nana, Arvind

Negoro, Hiromitsu

Neubert, Anne

Nguyen, Phi Duong

Nicholas, Joseph

Noel, Sabrina E

Nygaard, Kevin Heebøll

Obada, Bogdan

Ohana, Nissim

Oner, Ali

Onizuka, Naoko

Osawa, Yusuke

Özbaş, Nilgün

Özdemir, Ekrem

Ozden, Eyyup Sabri

Pan, Deyue

Pantouvaki, Anna

Paolo Rossi, Stefano Marco

Peay, Alice

Penfold, Rose S.

Poffenbarger, Mason

Princeton, Daisy Michelle

Protzuk, Omar

Qian, Qing

Qiao, Yusen

Rahmanipour, Elham

Rajha, Humam Emad

Ramadanov, Nikolai

Rao, Sudhaker D.

Ribbons, Karen

Ricciardi, Benjamin

Rittharomya, Jintana

Romeo, Nicholas

Roopsawang, Inthira

Ross, Jeremy

Rossettini, Giacomo

Rostagno, Carlo

Roytman, Gregory

Sadagatullah, Abdul Nawfar

Sağlam, Sönmez

Sahiner, Zeynep

Sarwath, Sadia

Sathiyaseelan, Naveen

Satpathy, Jibanananda

Savio, Sherly

Scaramuzzo, Laura

Schmucker, Abigail

Sharma, Shivam

Shen, Guohang

Shipway, David

Sidhu, Gur-Aziz Singh

Sivritepe, Ridvan

Smeeing, Diederik

Sorich, Megan

Stambough, Jeffrey

Stella, Alex Buoite

Stern, Brocha Z.

Suparb, Aree-Ue

Syamala, Shirmila

Szulc, Pawel

Takegami, Yasuhiko

Talerico, Michael

Tamura, Shuntaro

Tarantino, U.

Taşkın, Öztürk

Tedeschi, Roberto

Tian, Yun

Tong, Cindy

Tran, Bryant

Trujillo, Xochitl

Tsuge, Takahiro

Tunçez, Mahmut

Unver, Bayram

Uzel, Kadir

Van Bremen, Hanne-Eva

Vural, Abdussamed

Wang, Hao-Ming

Wang, Jichuan

Wang, Renkai

Wang, Xin

Weinschenk, Robert C.

Williamson, Frances

Wong, Khai Cheong

Wu, Cheng-En

Xia, Liang

Xiong, Liming

Yadala, Shravan Kumar

Yagi, Masahide

Yalın, Mustafa

Yamamoto, Norio

Yee, King Hang

Yeh, Kuang-Ting

Yeung, Meredith

Yiğit Gökmen, Mehmet

Yijia, Bryan Tan

Yokoo, Suguru

Yu, Xiang

Zhang, Jun

Zhang, Yuan-Wei

Zheng, Liujie

